# Miller-Fisher syndrome with positive anti-GD1b and anti-GM1 antibodies combined with multiple autoimmune antibodies: A case report

**DOI:** 10.1097/MD.0000000000034969

**Published:** 2023-08-25

**Authors:** Limei Zhang, Linqing Ma, Lihua Zhou, Lu Sun, Chunru Han, Qi Fang

**Affiliations:** a Department of Neurology, the People’s Hospital of Suzhou New District, Suzhou, Jiangsu, China; b Department of Neurology, the First Affiliated Hospital of Soochow University, Suzhou, Jiangsu, China.

**Keywords:** A/Ro52 antibody, anti, anti, anti, Fisher syndrome, ganglioside antibodies, Miller, SS, thyroid peroxidase antibody

## Abstract

**Rationale:**

Anti-ganglioside antibodies (AGA) play an essential role in the development of Miller-Fisher syndrome (MFS). The positive rate of ganglioside antibodies was exceptionally high in MFS, especially anti-GQ1b antibodies. However, the presence of other ganglioside antibodies does not exclude MFS.

**Patient concerns:**

We present a 48-year-old male patient who suddenly developed dizziness, visual rotation, nausea, and vomiting accompanied by unsteady gait and diplopia for 3 days before presentation to our clinic.

**Diagnoses:**

On physical examination, the patient’s right eye could not fully move to the right side and horizontal nystagmus was found. Coordination was also impaired in the upper and lower extremities with dysmetria and dysdiadochokinesia. The electromyography and cerebrospinal fluid examination results were normal. The serum anti-GQlb antibody test results were negative. However, serum anti-GD1b IgM and anti-GM1 IgM antibodies were positive. Meanwhile, the anti-thyroid peroxidase antibody was >600.00 IU/mL (0.00–34.00), and the anti-SS-A/Ro52 antibody was positive. He was diagnosed with MFS.

**Interventions:**

The patient received IVIg treatment for 5 days (0.4 g/kg/day) from day 2 to day 6 of hospitalization. On the 7th day of admission, the patient was administered intravenous methylprednisolone (500 mg/day), which was gradually reduced.

**Outcomes:**

The patient’s symptoms improved after treatment with immunoglobulins and hormones.

**Lessons:**

We report a case of MFS with positive anti-GD1b and anti-GM1 antibodies combined with multiple autoimmune antibodies. Positive ganglioside antibodies may be used as supporting evidence for the diagnosis; however, the diagnosis of MFS is more dependent on clinical symptoms.

## 1. Introduction

Miller-Fisher syndrome (MFS) is a variant of the Guillain-Barre syndrome (GBS).^[1]^ The typical triad is ocular paralysis, ataxia, and weakened or absent tendon reflexes.^[2]^ Notably, some patients develop drooping eyelids or pupil dilation. However, the pupillary response to light is normal.^[3]^ Additionally, some patients develop bulbar paralysis and/or facial muscle weakness.^[4]^ Some patients may also have numbness, hypesthesia in the distal limbs and face, and bladder dysfunction.^[5,6]^ Moreover, the onset of MFS is acute and can occur in any age or season. Furthermore, there is often a history of infection before onset, but sometimes, there may be no apparent cause.

Anti-ganglioside antibodies play an essential role in the development of MFS^[7]^ and anti-GQ1b antibodies have a higher positivity rate in these patients.^[8]^ A patient with MFS was admitted to our department with ophthalmoplegia, ataxia, and disappearance of tendon reflex. Anti-GQ1b antibody was negative, but anti-GD1b IgM and anti-GM1 IgM were positive. Anti-thyroid peroxidase antibody and anti-SS-A/Ro52 antibody were also positive. This report summarizes the clinical data of this patient and reviews the relevant literature to provide references for the early diagnosis of this type of patient.

## 2. Case presentation

A 48-year-old male patient was admitted to our department with dizziness and double vision that started 3 days prior to admission. He suddenly developed dizziness, visual rotation, nausea, and vomiting, accompanied by unsteady gait and diplopia for 3 days before his presentation to the clinic. He had no unconsciousness, difficulty speaking or swallowing, drooping eyelids, limb numbness, weakness, or fluctuating skeletal muscle weakness. The patient had no recent upper respiratory tract or gastroenteritis symptoms. Moreover, he denied exposure to recent immunization. He had a history of hypertension and denied a history of drug abuse or toxic exposure. Furthermore, he had no family history of genetic diseases.

The patient had normal body temperature and was mentally alert upon admission. On physical examination, his right eye could not fully move to the right side and horizontal nystagmus was found. His pupils were equally symmetrical, with an appropriate response to light. No facial droop or deviation of the tongue or uvula was observed. The patient gag reflex was normal. He could move all 4 extremities spontaneously. Diminished knee and ankle reflexes were bilaterally observed. The sensation was grossly intact in the face, trunk, and extremities. Coordination was also impaired in the upper and lower extremities with dysmetria and dysdiadochokinesia. The patient was unable to walk well and his bilateral flexor plantar responses were normal. Moreover, he had no visible involuntary movements, and his neck was supple, without signs of meningismus. Furthermore, rectovesical sphincter function was normal.

The patient symptoms did not change significantly on the second day after admission. Additionally, no abnormalities were observed on brain magnetic resonance imaging (MRI). The lumbar penetration pressure was 140 mmH_2_O. Cerebrospinal fluid (CSF) analysis revealed a white blood cell count of 2 × 10^6^/L and 328mg/L of protein. The patient received intravenous high-dose immunoglobulin (IVIg) treatment for 5 days (0.4 g/kg/day) from day 2 to day 6 of hospitalization. Serum antibodies were measured using an enzyme-linked immunosorbent assay for gangliosides (anti-GQ1b, GT1b, GD1b, GD1a, GM3, GM2, GM1 IgG and IgM), and the results were anti-GD1b IgM(+) and anti-GM1 IgM(++). All CSF antibodies tested negative for gangliosides. On the 7th day of admission, the patient was administered intravenous methylprednisolone (500 mg/day), which was gradually reduced. The patient was discharged with a significant improvement in symptoms on the 20th day of admission. He was administered prednisone orally and the prednisone dose was gradually reduced. Furthermore, the patient recovered completely after 1 month.

Laboratory test results, including routine blood, urinary, and fecal examinations, coagulation function, blood glucose, blood lipid, serum electrolytes, kidney function, liver function, homocysteine, tumor markers, and HBA1c, were unremarkable. Serological tests were negative for human immunodeficiency virus, syphilis, hepatitis B, and hepatitis C. Thyroid function showed FT4 was 24.69 pmol/L (12.00–22.00), TSH was 9.11 mIU/L (0.27–4.20), anti-thyroid peroxidase antibody was over 600.00 IU/mL (0.00–34.00), anti-thyroglobulin antibody was 95.99 IU/mL (0.00–115.00), thyrotropin receptor antibody was 2.180 IU/L (0.000–1.750), and anti-thyroglobulin antibody was 0.41 ng/mL (3.50–77.00). Anti-nuclear antibody examination showed positive results for the anti-SS-A/Ro52 antibody. CSF analysis (on the second day of admission) revealed a lumbar puncture pressure was 140 mmH_2_O, white blood cell count of 2 × 10^6^/L, protein level of 328 mg/L, chlorine was 125.1 mmol/L, and sugar was 3.35 mmol/L. Moreover, CSF viral serology, Gram staining, and culture were negative. There were also no abnormalities on the MRI. Chest computed tomography showed no apparent abnormalities. Electrocardiogram findings were normal. Cardiac ultrasonography revealed no abnormalities. Nerve conduction studies were normal, including sensory nerve action potentials and sensory nerve conduction velocity. H-reflex and F-wave were normal. Furthermore, no obvious abnormalities were observed on the electroencephalography.

## 3. Discussion

MFS is an acute immune-mediated polyneuropathy with a unipolar course, and is considered a subtype of GBS. It is characterized by ataxia, ophthalmoplegia, and areflexia.^[1,2]^ There are also incomplete forms, including acute ataxia without extraocular paralysis and acute extraocular paralysis without ataxia.^[9]^ Notably, some patients with MFS have limb weakness.^[10]^ Limb weakness is a primary symptom of GBS. These patients were diagnosed with GBS.^[4]^ The diagnosis of MFS mainly depends on clinical manifestations. A history of pre-disease infection, albuminocytologic dissociation,^[11]^ and positive anti-GQ1b antibodies are the primary supporting conditions for diagnosis. Anti-GQ1b antibodies are positive in a majority of MFS patients.^[12]^ Additionally, anti-GQ1b antibodies are positive in some Bickerstaff brainstem encephalitis (BBE) and GBS patients.^[13]^ Brain imaging can also be used to distinguish MFS from other diseases.

No prodrome infection or vaccination was observed in this patient. The initial symptoms were dizziness and double vision. Extraocular muscle paralysis, disappearance of the tendon reflex, and ataxia were observed during physical examination. There were no unconsciousness or pyramidal signs during the disease. MRI of the brain revealed no abnormal lesions, especially within the brainstem. No albuminocytologic dissociation was observed within 1 week of onset. Lumbar puncture was not performed again; therefore, whether albuminocytologic dissociation occurred 2 to 3 weeks after the onset could not be determined. Studies have also shown that some patients with MFS do not exhibit albuminocytologic dissociation in CSF.^[14]^ Additionally, some patients had slightly increased white blood cell counts, but decreased protein levels. Furthermore, electromyography revealed no signs of peripheral nerve damage, suggesting that the patient had no GBS.

MFS and BBE are assumed to be closely associated with serum anti-GQ1b antibodies and to share common immunological profiles. Diplopia and/or ataxia are the common initial clinical symptoms of both diseases. Tendon reflex is weakened or disappears in patients with MFS. There was no disturbance of consciousness. Consciousness disturbances and/or positive pyramidal signs, such as hypertendon reflexes and positive pathological signs, are found in patients with BBEs.^[15]^ On the basis of these symptoms, the patient was diagnosed with MFS. Studies have shown that the positivity rate of anti-GQ1b antibodies in serum ganglioside antibodies was higher in both groups.^[12,13]^

Gangliosides are widely distributed on the outer surface of the cell membranes in all tissues. They are also involved in the construction and stability of the cell membranes. It is also abundantly present in the nervous system. Anti-ganglioside antibodies play an essential role in the occurrence of GBS by targeting and attacking neuronal cells to induce GBS through immune mediation. Different subtypes of GBS ganglioside antibodies have certain specificity.^[7]^ Antibodies to gangliosides are also found in diseases such as motor neuron disease,^[16]^ myasthenia gravis,^[17]^ and Alzheimer disease.^[18,19]^ Furthermore, ganglioside antibodies, a subtype of GBS, play an essential role in MFS development.

Some scholars have proposed the concept of “anti-GQ1b antibody syndrome.” Anti-GQlb antibodies can bind to GQlb, which is highly expressed in the oculomotor, trochlear, abducens, and limb spindles, resulting in extraocular paralysis and ataxia. Simultaneously, it can also combine with the GQlb in the brainstem, causing a disturbance in consciousness. There are 6 types of anti-GQ1b syndromes: typical Miller Fisher syndrome, incomplete Miller Fisher syndrome, GBS, BBE, pharyngoneck brachial weakness, and overlap of different types. The diagnosis of anti-GQ1b antibody syndrome depends on the presence of anti-GQ1b IgG antibodies.^[9,20]^ The anti-GQ1b antibody was negative in our case, but the anti-GD1b IgM and anti-GM1 IgM were positive. As mentioned above, anti-GQ1b is positive with MFS patients. Patients with anti-GD1b and anti-GM1 antibody positivity or anti-GQ1b antibody negativity are rare. It has also been reported that a patient with multifocal motor neuropathy has both anti-GD1b and anti-GM1 antibodies.^[21]^ Therefore, the role of ganglioside antibodies in MFS pathogenesis requires further investigation. In our opinion, positive ganglioside antibodies can be used as supporting evidence for the diagnosis of MFS but cannot be used as an exclusion criterion. Furthermore, the diagnosis of MFS depends more on clinical symptoms.

The patient also tested positive for multiple autoimmune antibodies, including anti-thyroid peroxidase and anti-SS-A/Ro antibodies. Autoimmune diseases can involve a variety of autoimmune antibodies, and a patient with systemic lupus erythematosus was found to have GBS as the first symptom.^[22]^ Clinical symptoms occur only when a specific antibody destroys target tissue to a certain extent. Otherwise, there may be no clinical manifestation. The presence of multiple autoimmune antibodies may indicate more severe immune abnormalities in patients who are at a higher risk of recurrence. However, their clinical significance requires further investigation.

In summary, although the positivity rate of ganglioside antibodies is exceptionally high in MFS, especially anti-GQ1b antibodies, the presence of other types of ganglioside antibodies does not exclude MFS. Positive ganglioside antibodies may be used as supporting evidence for diagnosis rather than relying solely on their positive status as a basis for diagnosis. Notably, the diagnosis of MFS is dependent on the clinical symptoms.

## Acknowledgments

We are thankful to the patient for his participation in this study.

## Author contributions

**Conceptualization:** Limei Zhang.

**Formal analysis:** Lu Sun.

**Funding acquisition:** Linqing Ma.

**Investigation:** Limei Zhang.

**Methodology:** Limei Zhang.

**Project administration:** Limei Zhang, Linqing Ma.

**Resources:** Limei Zhang.

**Supervision:** Linqing Ma, Qi Fang.

**Validation:** Lihua Zhou, Lu Sun, Chunru Han.

**Visualization:** Lihua Zhou, Lu Sun, Chunru Han.

**Writing – original draft:** Limei Zhang.

**Writing – review & editing:** Linqing Ma.
